# Erectile Dysfunction Is Common after Rectal Cancer Surgery: A Cohort Study

**DOI:** 10.3390/curroncol30100673

**Published:** 2023-10-20

**Authors:** Sebastian Borgund Hansen, Birthe Thing Oggesen, Siv Fonnes, Jacob Rosenberg

**Affiliations:** 1 Center for Perioperative Optimization, Department of Surgery, Herlev and Gentofte Hospital, University of Copenhagen, Borgmester Ib Juuls Vej 1, DK-2730 Herlev, Denmarkjacob.rosenberg@regionh.dk (J.R.); 2 The Late-Complication Clinic, Capital Region of Denmark, Department of Surgery, Herlev and Gentofte Hospital, University of Copenhagen, Borgmester Ib Juuls Vej 1, DK-2730 Herlev, Denmark

**Keywords:** rectal neoplasms, erectile dysfunction, survey

## Abstract

Erectile dysfunction is a known late complication following surgery for rectal cancer. We aimed to determine the prevalence of erectile dysfunction after rectal cancer surgery and characterize it. This was a prospective observational cohort study. Data from men after surgery for rectal cancer were collected between October 2019 and April 2023. The primary outcome was the prevalence of erectile dysfunction following surgery based on the International Index of Erectile Function questionnaires, IIEF-5 and 15. Secondary outcomes were prevalence in subgroups and self-perceived erectile function. In total, 101 patients agreed to participate, while 67 patients (67%) responded after a median six-month follow-up after surgery. Based on IIEF-15, 84% of the patients had erectile dysfunction. For subgroups, 74% of patients who underwent robot-assisted surgery had erectile dysfunction, whereas all patients who underwent either laparoscopic or open surgery had erectile dysfunction (*p* = 0.031). Furthermore, half of the patients rated their self-perceived ability to obtain and keep an erection as very low. In conclusion, in our cohort, erectile dysfunction was common after rectal cancer surgery, and half of the patients were unconfident that they could obtain and keep an erection. Information regarding this finding should be given so that patients feel comfortable discussing therapeutic solutions if needed.

## 1. Introduction

Rectal cancer has a global annual incidence of 700,000 cases [1]. Better surgical, oncological, and perioperative care has increased cancer survival [2,3]. This has led to a growing risk of late complications, including the risk of erectile dysfunction [4,5]. Surgery, chemo- and radiotherapy are cornerstones in the treatment of rectum cancer and in the prevention of local recurrence and metastatic disease. Surgical resection of the rectum poses a risk of intraoperative nerve damage to the pelvic plexus nerves, which has been suggested to be the primary reason for postoperative sexual dysfunction [3]. Moreover, the extent of surgery varies based on the tumor’s proximity to the anus and the local spread of the cancer, necessitating varying degrees of extensive surgery in the lesser pelvis [6], and surgical technique may therefore play a role. Chemo-radiation therapy might also pose an independent risk for erectile dysfunction because of damage to nerves and vessels [7].

This prospective cohort study aimed to determine the prevalence of erectile dysfunction in male patients following surgery for rectal cancer in a Late-Complication Clinic. Furthermore, we investigated the prevalence of erectile dysfunction in subgroups consisting of different surgical approaches and chemoradiotherapy strategies, as well as the self-perceived ability to obtain and keep an erection.

## 2. Materials and Methods

This study was a prospective, observational, single-center cohort study. This study was reported in accordance with the STrengthening the Reporting of OBservational studies and Epidemiology (STROBE) guidelines for cohort studies [8]. The study population consisted of patients attending the Late-Complication Clinic [4] at Herlev Hospital, Denmark, which is a tertiary university hospital in Denmark, covering 10% of the national population as catchment area. Data were collected from October 2019 to April 2023. All patients who underwent rectal cancer surgery were invited to the Late-Complication Clinic at Herlev Hospital, where patients can be treated for late complications that might occur after surgery. The patients invited to the Late-Complication Clinic and included in this study were unselected since all patients who underwent surgery were invited and represented a sample from the average community. Patients were invited to the Late-Complication Clinic and agreed and consented to answer electronic Patient-Reported Outcome Measures (ePROMs) after surgery [4]. We included all patients who had answered the ePROM before 30 April 2023. Questionnaires were subsequently sent to the patients either by mail or via their E-boks [9], which is the personal, secure online Danish digital mailbox linked to each resident through their unique personal identity number.

Eligible patients were men with cancer of the rectum who underwent surgical treatment. Patient data were collected from electronic medical records (e.g., histopathological and surgery reports) and entered in Research Electronic Data Capture (REDCap) [10]. Data from questionnaires were filled out at home by patients directly in REDCap. Patient characteristics were collected from electronic medical records and consisted of age (in years), American Society of Anesthesiologists-score (ASA-score) [11], diagnosed diabetes mellitus II (DMII), and Body Mass Index [12] (BMI in kg/cm^2^). With regards to the cancer variables, data were collected from (1) histopathology reports including tumor, node, and metastasis stage (TNM); and (2) surgical reports including the length of the tumor from the anus in centimeters, as well as data concerning surgical approaches (open, laparoscopic, conversion from laparoscopic to open, robot-assisted, and endoscopic/endoluminal techniques). Data concerning neoadjuvant chemoradiotherapy, defined as chemo- or radiotherapy prior to surgery; adjuvant therapy, defined as chemo- or radiotherapy after surgery; or no neoadjuvant or adjuvant therapy, were collected from electronic medical records. The degree of erectile dysfunction was evaluated through the International Index of Erectile Function (IIEF-15) in a Danish-validated version, which is a cross-culturally and psychometrically valid measure of erectile dysfunction in males [13]. This questionnaire is composed of 15 questions (Q1–15) and five domains: erectile function (Q1–5, 15), orgasmic function (Q9, 10), sexual desire (Q11, 12), intercourse satisfaction (Q6–8), and overall satisfaction domain (Q13, 14). The erectile function domain of the IIEF-15 questionnaire has been validated as a diagnostic tool in the clinical setting for grading degrees of severity of erectile dysfunction and for distinguishing between men with and without erectile dysfunction [14,15]. Through the IIEF-15, erectile dysfunction can be classified into groups based on the Erectile Function (EF) domain as follows: no erectile dysfunction (EF score 26–30), mild erectile dysfunction (EF score 22–25), mild to moderate (EF score 17–21), moderate (EF score 11–16), and severe erectile dysfunction (6–10) [13]. The International Index of Erectile Function-5 (IIEF-5), also known as the Sexual Health Inventory for Men (SHIM), consists of questions 2, 4, 5, 7, and 15 from the IIEF-15. The IIEF-15 questionnaire has a cut-off value for erectile dysfunction at a score of <26, while the IIEF-5 has a cut-off at a score of 21 points, dividing patients dichotomously, with patients scoring equal to the cut-off or below as having erectile dysfunction [15,16].

Statistical analyses were performed in R (version 4.1.0; R Foundation for Statistical Computing, Vienna, Austria). Normality was assessed with QQ plots and histograms to evaluate if the continuous data were normally distributed. Continuous data were presented as median and interquartile range if not normally distributed or mean and standard deviation if normally distributed. With regards to categorical data, both ordinal categories, such as BMI categories, ASA-score, and TNM, and nominal categories, such as surgical approach, were reported in numbers and percentages. Regarding missing data, patients were excluded if they failed to answer all the questions of the IIEF-15 and IIEF-5 used to calculate the erectile dysfunction rate. The same applied to missing surgery descriptions or data from patients’ files. In all tests, *p* ≤ 0.05 was considered statistically significant. Explorative analyses, such as the subgroup analysis of erectile dysfunction (different surgical approaches and chemoradiotherapy strategies), did not fit parametric statistics. They were examined with a chi-square test or Fisher’s exact test. The subgroup analysis on erectile dysfunction was calculated if more than five patients had the specific treatment. The surgical approaches of open surgery, conversion, and laparoscopic were grouped together in the subgroup analysis, while only one patient had endoscopic (endoluminal) surgery and hence was excluded.

Approval from the Danish Data Protection Agency was obtained (P-2020-134). During the establishment of the Late-Complication Clinic, the Regional Committee on Health Research Ethics was contacted and confirmed that the project was exempt from formal ethics committee approval (20033634). Patients were informed about the survey, and consent was obtained from all eligible patients.

## 3. Results

**Figure 1:**
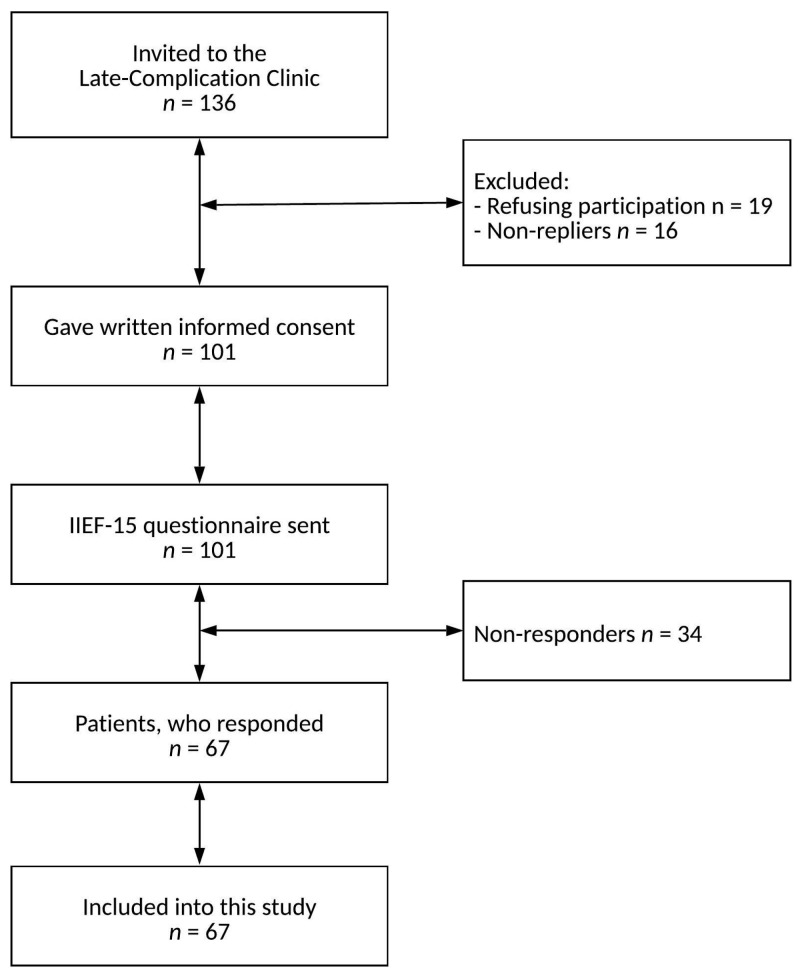
Flow diagram of patient inclusion.

Of the 136 male patients approached, 101 agreed to answer the ePROMs; see Figure 1.

There were 67 patients who responded to the questionnaire, yielding a response rate of 67%. Five patients failed to complete at least one question in the erectile function domain on the IIEF-15 or a question in the IIEF-5 and were thus excluded. Patient characteristics are shown in Table 1. The men had a median age of 67 years, their median BMI was 25 kg/m^2^, and 12% of them had DMII. The median time to follow-up was six months after surgery. The most frequent surgical approach was robot-assisted surgery, accounting for 64% of the operations.

With regards to chemoradiotherapy, 27% of the patients received neoadjuvant chemotherapy, 34% neoadjuvant radiotherapy, and 24% adjuvant chemotherapy. The most frequent tumor stage, according to the TNM classification, was T3 in 45% of the patients, N0 in 67%, and M0 in 91%. The tumor’s median length from the anus was 8 cm.

The scores from the IIEF-15 questionnaire are presented in Table 2.

Erectile dysfunction was found in 52 of 62 patients; thus, 84% of the patients in both the IIEF-15 (Table 2) and IIEF-5 (Table 3) questionnaires.

**Figure 2:**
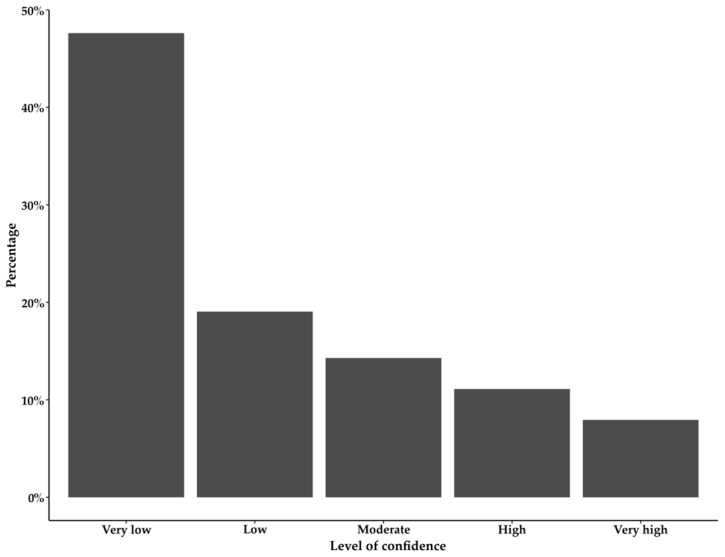
Bar chart showing the distribution in percentage on the y axis of the 63 answers to question 15 from the International Index of Erectile Function 15 [13]: “How do you rate your confidence that you could obtain and keep an erection?”.

In general, the men had low scores on the domains regarding orgasmic function and intercourse satisfaction, where half of the men had a median score of 0 and 1, respectively. However, the median score of the sexual satisfaction domain was 5; see Table 2. Self-perceived confidence (Q15) in obtaining an erection was rated as very low by 30 of 62 patients, accounting for 48% (Figure 2).

The subgroup analysis of the prevalence of erectile dysfunction is presented in Table 4.

With regards to different surgical approaches, 74% of the patients having robot-assisted surgery had erectile dysfunction, while all patients having either laparoscopic or open surgery had erectile dysfunction (*p* = 0.031). No obvious correlation was found between the choice of surgical approaches and the T-stage). Concerning chemoradiotherapy, erectile dysfunction was present in 69% of the patients who solely received chemotherapy, 81% of the patients who did not receive any chemoradiotherapy, and 95% of the patients who received radiotherapy. No statistical difference was found between patients receiving radiotherapy and patients who either did not receive any treatment or who only had chemotherapy (*p* = 0.076).

## 4. Discussion

Approximately five out of six men had erectile dysfunction at a median of six months after rectal cancer surgery, and half of the patients reported their self-perceived confidence to obtain and keep an erection as “very low”. The prevalence of erectile dysfunction in the subgroup of patients who underwent robot-assisted surgery might be lower than in patients undergoing other surgical approaches.

We found that 84% of the patients had erectile dysfunction at a median of six months after surgery for rectal cancer measured with IIEF-15 and IIEF-5. Other retrospective studies have found similar rates of erectile dysfunction, ranging from 80 to 89% [5,17], with median follow-up periods up to 21 months after surgery [5]. However, erectile function could improve over time. Another prospective study found that 87% had erectile dysfunction one month after surgery, improving to 72% after 12 months [18]. However, it is unclear if this improvement was due to an intervention or a natural recovery. We are continuously gathering data on patients in the Late-Complication Clinic to determine whether the erectile function of the patients show signs of natural recovery, medically aided improvement, or whether the erectile dysfunction is stationary. In a survey of the background population consisting of 21,394 Danish men, 11% of the 65–74-year-old men had moderate or severe erectile dysfunction [19]. Thus, erectile dysfunction has a higher prevalence in patients operated on for rectal cancer than the Danish background population, with whom we assume our population is comparable. However, we did not collect data regarding erectile dysfunction prior to surgery, so caution must be exercised with regard to drawing conclusions. In our study, the patients positioned themselves on the extremes of the IIEF-5 scale. There were 16% of the patients who did not have erectile dysfunction, while 76% of the patients reported severe erectile dysfunction. It is very interesting to investigate this pattern of data in other cohorts to uncover if this reflects the distribution of data in other populations as well. This would help verify whether the observed distribution is a valid reflection of the clinical situation for this patient group. Otherwise, these extreme answers could be caused by a non-responder bias in our cohort. A non-responder bias could lead to men with only a minor decrease in their erectile function did not respond to the questionnaire, while the patients who either did not have erectile dysfunction or who had severe dysfunction found it important to respond. However, as our response rate was 67%, the influence of non-responder bias on our data should be limited. Nevertheless, we cannot definitively ascertain this until the data patterns in other studies have been thoroughly evaluated, all while taking into account the response rates. As secondary outcomes, we explored whether different surgical approaches or chemoradiotherapy had an impact on the prevalence. Erectile dysfunction was present in 78% of the patients who underwent robot-assisted surgery and in 100% of the patients who underwent either laparoscopic or open surgery. Because our cohort is relatively small, our findings should be carefully interpreted. The relationship between different surgical approaches’ impact on erectile dysfunction has also been examined in another study. A prospective study of 135 patients who underwent one of two types of robot-assisted or laparoscopic surgery reported a decrease in IIEF-15 scores across all arms after surgery [20], with scores improving after 12 months. However, the patients who underwent robot-assisted surgery appeared to have a faster recovery rate [20]. In our study, robot-assisted surgery accounted for 64% of the operations, but because the data are sparse, we cannot draw any firm conclusions. With regards to chemoradiotherapy, 95% of patients who received radiotherapy had erectile dysfunction. However, there was no statistical difference in erectile dysfunction compared with patients who did not receive any radiotherapy. This is important because an increasing number of patients are treated without surgery but merely with radiation and chemotherapy. One study found that patients who received curative radiotherapy without surgery also experienced erectile dysfunction following treatment [7], and a prospective study including 201 men undergoing surgery for rectal cancer found that radiotherapy appeared to have an adverse effect on male sexual function in addition to any impairment resulting from surgery alone [21].

We found that almost half (48%) of the patients had very low self-perceived confidence to obtain and keep an erection. In the survey of the background population [19], sexual dysfunction concerning erectile dysfunction was perceived as a problem always or often in one in four (25%) of the 64–74-year-old men. This gap between the self-perceived confidence to obtain and keep an erection and it being perceived as problematic could be due to that 32% of 65–74-year-old men are sexually inactive [19]. While the self-perceived ability to obtain an erection and perceiving erectile dysfunction are similar, they are not identical, and the sudden nature of cancer treatment might address why fewer men in the background population may see it as a problem since sexual well-being is subjective. However, while the median score of the intercourse satisfaction domain was 0, the median score of the sexual desire domain was 5, which might indicate an unfulfilled desire to take part in a sexual relationship, thus pointing to a decreased quality of life.

A strength of this study was the prospective design, thus limiting the risk of recall bias. Furthermore, we used both a validated data collection method and validated questionnaires. The reporting of this cohort study was according to the STROBE [8] guideline, which ensures transparency in the reporting. This study was based on original data, and the hospital covers 10% of the country as an unselected catchment area, which ensures the generalizability of our result. We had a response rate of 67%, which is acceptable in terms of limiting non-response bias [22]. One limitation of this study is the limited number of included patients (67 cases), which is in part due to its single-center design. This can limit our statistical strength for detecting differences between subgroups, such as variations between different surgical techniques or with different chemoradiotherapy treatment regimens. Limitations also included a median time to the first response of six months. Ideally, this should have been three months since the patients of the Late-Complication Clinic received questionnaires 3, 6, 12, 24, and 36 months after their surgery date. We did not collect data on erectile dysfunction prior to surgery, and many of the men were not sexually active at follow-up. However, it is difficult to determine whether they were inactive because of erectile dysfunction or because of other reasons.

This study is highly clinically relevant as it can be used as a base for better patient guidance and information for late complications since sexual function is regarded as a core outcome in rectal cancer surgery [23] and, therefore, must be of importance to the patients. PROMs such as the IIEF-15 or IIEF-5 are obviously important to unlock the potential of value-based healthcare [24]. Moreover, suppose more detailed information concerning frequent postoperative late complications is available for patients, e.g., in patient information brochures. In that case, they might feel more comfortable discussing their postoperative erectile dysfunction with their healthcare providers so that possible therapeutic treatment can be explored. It is possible that an affected erectile function following rectal cancer surgery can improve over time [18]. However, the dynamic nature of erectile function recovery is not well understood yet. It is indeed a critical aspect of this topic, and some studies have proposed a natural recovery following rectal cancer surgery with a recovery time of up to one year from the date of surgery [18,20]. Others are advocating for early penile rehabilitation, defined as any intervention or combination with the goal not only to achieve erections sufficient for satisfactory sexual intercourse but also to return erectile function to preoperative levels [25]. Various strategies have been explored to enhance and expedite the improvement of erectile function in patients after surgical treatment, such as the administration of phosphodiesterase-5 inhibitors such as tadalafil or sildenafil. A systematic review including 253 patients found that postoperative administration of phosphodiesterase-5 inhibitors appeared to improve the IIEF scores in the short term in male patients diagnosed with erectile dysfunction following surgery for rectal cancer. However, the literature is sparse, necessitating further studies to address the natural course of the situation as well as the long-term efficacy of treatment and alternative strategies [26]. Therefore, there is a need for further follow-up of individual patients with erectile dysfunction over time to evaluate whether there is a natural recovery, as indicated in a previous study [20], or if penile rehabilitation efforts are needed. It is essential to explore safer and more effective treatments for rectal cancer and their relationship to late complications. Our study has offered some insight into differences amongst a variety of treatment regimens and surgical techniques, although the number of patients included in this study prevents any certain conclusions regarding the prevalence of erectile dysfunction after different surgical and oncological treatment regimens. However, the feasibility of discussing various surgical regimens is constrained by the clinical challenges posed by the presence of large or locally advanced tumors necessitating more extensive surgical interventions, although this should be a focus of future studies. From the results of this study, one can only conclude that erectile dysfunction is indeed a very common condition following rectal cancer surgery and that future studies should examine to what extent this is a concern for the patients, considering the patients’ specific course of treatment for their rectal cancer. Moreover, the patients in the Late-Complication Clinic, who were recruited for the purpose of studying the prevalence of erectile dysfunction, are being followed up for up to 36 months after their surgery. Thus, the process for recovery or worsening of erectile dysfunction could be a potential subject of further investigation and would be of utmost interest to the expanding field of late complications and patient-centered healthcare. There is probably a natural recovery of erectile dysfunction after surgery, and in future studies, we will follow our patients’ erectile function over time. If they report erectile dysfunction, we plan to offer them penile rehabilitation and evaluate how this affects the patients’ postoperative late complications regarding erectile dysfunction.

## 5. Conclusions

Approximately five out of six males reported erectile dysfunction following rectal cancer surgery. The prevalence of erectile dysfunction might be lower in the patients who had robot-assisted surgery compared with other surgical approaches, and no difference was found between patients who received radiotherapy and those who did not. Patients should be informed in detail preoperatively that erectile dysfunction is common so that they feel comfortable discussing therapeutic solutions. Future studies should focus on exploring preventive measures to reduce the risk of erectile dysfunction.

## Figures and Tables

**Table 1:** Patient characteristics. Data are given as numbers (%) if not stated otherwise.

Patients’ Characteristics at Surgery		Responders (*n* = 67)
Age in years, mean ± SD		67 ± 11
Diabetes Mellitus Type II		8 (12)
BMI kg/cm^2^, median (IQR)		25 (23–27)
	<18.5	3 (4)
	18.5–25	34 (51)
	25–29.9	19 (28)
	30–34.9	9 (13)
	35–39.9	2 (3)
	<40	0 (0)
ASA class I–II		51 (76)
ASA class III–IV		16 (24)
Tumor classification		
	T0	6 (9)
	T1–2	25 (39)
	T3	29 (45)
	T4	4 (6)
Node classification		
	Not nodes evaluated	1 (2)
	N0	43 (67)
	N1–2	20 (31)
Metastasis classification		
	M0	58 (91)
	M1	6 (9)
Length from anus in cm, median (IQR) *		8 (6–11)
Surgical approach		
	Open surgery	6 (9)
	Laparoscopic surgery	15 (23)
	Transition to open surgery	1 (2)
	Robot-assisted surgery	41 (64)
	Endoscopic surgery	1 (2)
Chemo-/radiotherapy		
	Received neoadjuvant chemotherapy	18 (27)
	Received neoadjuvant radiotherapy	23 (34)
	No preoperative treatment	36 (54)
	Received adjuvant chemotherapy	16 (24)
	Received adjuvant radiotherapy	1 (1)
	No postoperative treatment	46 (69)

ASA: American Society of Anesthesiologists; BMI: Body Mass Index; IQR: Interquartile range; *n*: number; Neoadjuvant: treatment before surgery; Adjuvant: treatment after surgery. * Missing data due to tumor length from anal verge not mentioned in the surgical report (*n* = 61).

**Table 2:** Scores of the patients in the different domains of the International Index of Erectile Dysfunction 15 (IIEF-15) [13].

Domains of IIEF-15 (Score)	Question	Number	Median	IQR
IIEF erectile function (1–30)	1, 2, 3, 4, 5, 15	62	4	1–11
IIEF-15 erectile dysfunction (≤25) *	1, 2, 3, 4, 5, 15	52		
IIEF orgasmic function (0–10)	9, 10	64	1	0–8
IIEF sexual desire (2–10)	11, 12	65	5	3–7
IIEF intercourse satisfaction (0–15)	6, 7, 8	65	0	0–4
IIEF overall satisfaction (2–10)	13, 14	59	6	3–7

The minimal and maximum scores are shown in parentheses. The higher the score, the better the sexual function in each domain. IQR: interquartile range. * IIEF erectile function domain score ≤25 indicates erectile dysfunction [15].

**Table 3:** Erectile dysfunction scores of the 62 patients were based on the International Index of Erectile Function 5 (IIEF-5) questionnaire [16].

IIEF-5 Erectile Dysfunction (Score)	Number	%
Severe erectile dysfunction (1–7)	47	76
Moderate erectile dysfunction (8–11)	1	2
Mild to moderate erectile dysfunction (12–16)	3	5
Mild erectile dysfunction (17–21)	1	2
No erectile dysfunction (22–25)	10	16
Any form of erectile dysfunction (≤21)	52	84

The scoring range of severity is shown in parenthesis, with a higher score signifying better sexual function in each domain.

**Table 4:** The prevalence of erectile dysfunction according to the International Index of Erectile Function (IIEF-15) [15] in subgroups of patients according to surgical approach and chemoradiotherapy. The subgroup analysis was only performed if the total number in the grouping was more than five patients; hence, there are no data for the endoluminal surgical approach.

Subgroups		Total, *n*	Erectile Dysfunction, *n* (%)
Surgical approach			
	Robot-assisted	38	28 (74)
	Open and converted operations	7	7 (100)
	Laparoscopic	13	13 (100)
Chemoradiotherapy			
	No chemoradiotherapy before or after surgery	21	17 (81)
	Radiotherapy with/without chemotherapy	21	20 (95)
	Chemotherapy alone	16	11 (69)

## Data Availability

The data presented in this study are available on request from the corresponding author. The data are not publicly available due to Danish legislation.
